# Bat guilds, a concept to classify the highly diverse foraging and echolocation behaviors of microchiropteran bats

**DOI:** 10.3389/fphys.2013.00164

**Published:** 2013-07-03

**Authors:** Annette Denzinger, Hans-Ulrich Schnitzler

**Affiliations:** Animal Physiology, Institute for Neurobiology, University of TübingenTübingen, Germany

**Keywords:** bat, echolocation, guild, community structure, habitat, foraging behavior

## Abstract

Throughout evolution the foraging and echolocation behaviors as well as the motor systems of bats have been adapted to the tasks they have to perform while searching and acquiring food. When bats exploit the same class of environmental resources in a similar way, they perform comparable tasks and thus share similar adaptations independent of their phylogeny. Species with similar adaptations are assigned to guilds or functional groups. Habitat type and foraging mode mainly determine the foraging tasks and thus the adaptations of bats. Therefore, we use habitat type and foraging mode to define seven guilds. The habitat types open, edge and narrow space are defined according to the bats' echolocation behavior in relation to the distance between bat and background or food item and background. Bats foraging in the aerial, trawling, flutter detecting, or active gleaning mode use only echolocation to acquire their food. When foraging in the passive gleaning mode bats do not use echolocation but rely on sensory cues from the food item to find it. Bat communities often comprise large numbers of species with a high diversity in foraging areas, foraging modes, and diets. The assignment of species living under similar constraints into guilds identifies patterns of community structure and helps to understand the factors that underlie the organization of highly diverse bat communities. Bat species from different guilds do not compete for food as they differ in their foraging behavior and in the environmental resources they use. However, sympatric living species belonging to the same guild often exploit the same class of resources. To avoid competition they should differ in their niche dimensions. The fine grain structure of bat communities below the rather coarse classification into guilds is determined by mechanisms that result in niche partitioning.

## Diversity in bats

The order Chiroptera consists of 19 families including the Pteropodidae. The key character that distinguishes bats from all other mammals is the capacity of powered flight and in microchiropteran bats the use of a tonal echolocation system (Denzinger et al., 2004; Schnitzler et al., 2004; Jones and Teeling, 2006). Microchiropteran bats comprise about 1000 species and are one of the most diverse groups within terrestrial mammals. In the course of evolution, numerous adaptations in behavior and in sensory and motor systems allowed bats to radiate into a multitude of niches at night which are occupied by other animals during the day. Bats exploit a great variety of food sources including insects and other arthropods such as scorpions and spiders, fish, small vertebrates, fruit, nectar and pollen, and even blood. They forage for airborne prey, glean food items from the ground or from vegetation, or forage above water surfaces for insects or fish. Bats occupy all terrestrial areas with the exception of the polar region and high mountain ranges and even use extreme habitats, i.e., *Otonycteris hembrichii* feeding in the desert on scorpions, or *Myotis vivesi* living on small isolated islands and hunting for fish in the ocean.

## Aims of this study

To understand the factors which underlie the radiation of bats into so many different directions, we have to identify the mechanisms that structure the high diversity in bats. There have been many approaches to classify bats into groups that face similar constraints (for review see: Fenton, 1990; Kalko et al., 1996; Kalko, 1997; Schnitzler et al., 2003). Food and feeding mode was often used as a basis for categorization leading to feeding associations like aerial insectivory, foliage-gleaning insectivory, piscivory, sanguinivory, nectarivory, frugivory, omnivory, and carnivory (McNab, 1971; Hill and Smith, 1984). Wing morphology and diet have been also used to separate bats into groups like: fast hawking, slow hawking, trawling, gleaning and hovering, fly-catching and perch hunting (Norberg and Rayner, 1987). Patterns of habitat use and variations of this approach have been used to identify groups of bats with similar foraging behaviors (Aldridge and Rautenbach, 1987; Crome and Richards, 1988; Neuweiler, 1989; Fenton, 1990). Elisabeth Kalko, who is honored with this edition of Frontiers in Integrative Physiology developed—together with others—this habitat oriented approach further and arranged bats that live under similar ecological conditions and perform similar echolocation tasks into guilds or functional groups (Kalko et al., 1996; Schnitzler and Kalko, 1998, 2001; Schnitzler et al., 2003; Denzinger and Schnitzler, 2004). The aims of this paper are to critically discuss the studies which have used the guild concept for classification of microchiropteran bats, and to further refine this approach. We will examine whether the arrangement of bats in functional groups is suited to identify the driving forces which determine the organization of bat communities. With our work we also want to honor Björn Siemers, to whom this edition of Frontiers in Integrative Physiology is also dedicated. In his scientific work Björn Siemers investigated the role of sensory and cognitive abilities of bats for defining a species' niche. Here we will discuss his approach on niche partitioning in bats within the guild concept.

## The guild concept

Root (1967) defined a guild as “a group of species that exploit the same class of environmental resources in a similar way.” Bats belonging to different guilds should therefore differ in the environmental resources they exploit and/or in the way how they do this. The basic idea behind the guild concept is that bats performing the same tasks share similar adaptations. We will outline that the attribution of bats into functional groups or guilds helps us to understand the organization of the highly diverse microchiropteran bat communities.

## Basic echolocation tasks of foraging bats

Foraging bats continuously emit echolocation signals and analyze the sound complex consisting of the emitted signal and the returning echoes in their auditory system to perform the basic echolocation tasks: detection, localization and classification. For detection, bats have to decide whether they perceive echoes form their own emitted signals or not. For localization bats determine the target distance by measuring the time delay between the emitted signal and the echo, and the target direction by using binaural and monaural echo cues. For classification bats use echo features such as spectrum and modulation patterns which encode the nature of the reflecting target (Schnitzler and Kalko, 1998, 2001; Schnitzler et al., 2003).

All bats have to perform several tasks in parallel when searching for food:

### Spatial orientation

Bats need to know their own position in relation to the world around them. This self-positioning has two aspects: navigation and obstacle avoidance. Bats navigate from their roosts to their hunting grounds and back. Thus, they have the ability to find, learn and return to specific places (Trullier, 1997; Schnitzler et al., 2003; Thiele and Winter, 2005). Each identified target can serve as a potential landmark for orientation in space. Landmarks within the perceptual range of a bat are used for route planning and route following. For long-range navigation, however, other senses like vision and the magnetic sense must be used (Schnitzler et al., 2003; Holland et al., 2006, 2008; Wang et al., 2007). Background objects are physical structures which may influence the flight behavior of bats. The closer a bat forages to the background, the smaller the available space for food acquisition maneuvers, and the higher the collision risk. The sensory and motor problems of foraging under these restricted conditions are reflected in specific sensory and motor adaptations. Distance dependent changes in echolocation behavior in the vicinity of background targets suggest that bats collect information needed for flight path planning and for collision avoidance. Adaptations in wing morphology that increase maneuverability of the bats also help them to forage successfully in restricted spaces (Aldridge and Rautenbach, 1987; Norberg and Rayner, 1987; Fenton, 1990; Norberg, 1994).

### Biotope recognition

The properties and the composition of the environment are important information for bats. Typical foraging grounds like forest edges, trees and bushes, meadows, and water surfaces are indicators for specific prey. In other words, they are biotopes which provide specific resources. Therefore, biotope recognition is fundamental for bats. Bats can use statistical properties of echoes from vegetation for the classification of typical biotope elements such as trees and bushes (Yovel et al., 2009, 2011).

### Food finding

Foraging bats have to find food. The ability to detect, classify and localize a food item strongly depends on where the food item is positioned. An insect flying far from the bat in open air constitutes a different foraging task from an insect sitting on a leaf. For many bats species, echolocation delivers all information necessary to find the food. If echolocation is not sufficient sensory cues such as odor or prey-generated sounds are used to find food.

The three tasks—spatial orientation, biotope recognition and food finding—often have to be performed in parallel. For example, an oak tree may be an important landmark along the foraging route and at the same time may also be an obstacle which needs to be avoided. Additionally, it may be an indicator for specific prey which has to be identified.

The psychophysics of hearing limits the processing of echo information. The emitted signal produces a forward-masking effect if it overlaps with or is close in time to the food echo. The echoes from background targets or clutter echoes produce a backward-masking effect if they overlap with or are close to the food echo. These masking effects prevent or reduce the chance of finding food. Comparative studies in the field and in the laboratory revealed that bats tend to avoid overlap of the target echo with the emitted signal as well as with clutter echoes from background targets (Kalko and Schnitzler, 1989, 1993). An exception are bats that use CF-FM signals consisting of a long component of constant frequency (CF) followed by a shorter downward frequency modulated terminal component (FM). These bats tend to avoid an overlap of the FM component.

**Figure 1:**
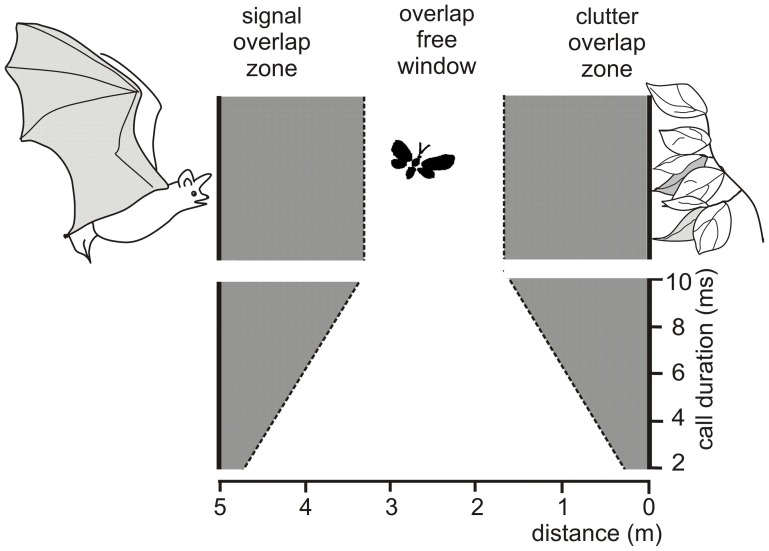
**Schematic drawing illustrating the conditions for overlap between emitted signal, prey echo and background echoes a bat encounters when foraging at a distance of 5 m to vegetation**. The prey echo overlaps with the emitted signal when an insect flies in the signal overlap zone and with the clutter echoes from the background when it flies in the clutter overlap zone. In the overlap-free window no overlap occurs. The width of the overlap zones depend on signal duration. At durations between 10 and 2 ms, the overlap zones range between 1.70 and 0.34 m, if a sound speed of 340 m/s is assumed. A reduction of signal duration by 1 ms reduces the width of each overlap zone by 0.17 m and thus increases the width of the overlap-free window by 0.34 m.

Due to the masking effects of the emitted signal and of the clutter echoes bats can only find food items without interferences if their echoes are positioned in the overlap-free window. This window is defined as the area between signal overlap zone where the emitted signal overlaps with the food echo and the clutter overlap zone where the food echo overlaps with clutter echoes from the background (Figure 1) (Kalko and Schnitzler, 1993; Schnitzler and Kalko, 1998, 2001; Schnitzler et al., 2003). The width of the signal and the clutter overlap zone depends on signal duration. For example, at an assumed sound speed of 340 m/s a signal duration of 10 ms produces an overlap zone which is 1.7 m wide. If undisturbed detection of a food item is only possible beyond the signal overlap zone, signal duration can be used as a rough measure for the minimal detection distance. Each increase of sound duration by 1 ms increases the width of the signal overlap zone and with it the minimal detection distance by 0.17 m. Sound duration also controls the width of the overlap-free window. A reduction of 1 ms widens the window by 0.34 m as it reduces each of the overlap zones by 0.17 m.

The degree of masking also depends on the frequency structure and on the SPL of the interfering signals and decreases with increasing steepness of a signal (Schnitzler et al., 2003). Thus, the masking zone can be smaller than the overlap zone calculated from signal duration if bats use signals which are more masking-tolerant. For example, *Myotis nattereri* use steeply modulated signals of large bandwidth which tolerate some overlap between prey and clutter echoes (Siemers and Schnitzler, 2000) (Figure 6). All bats using long CF-FM signals have solved the masking problem in a different way: They compensate for Doppler shifts and keep the target echo of the CF component in the extremely sharply tuned neurons of their auditory fovea whereas the CF component of the emitted signal has a lower frequency and falls in an area where the auditory threshold is high (Schnitzler and Denzinger, 2011). Therefore, masking of the CF component is prevented.

## Foraging habitats and foraging modes

Comparative studies showed that the distance between bat and background or food and background is the most relevant ecological condition for foraging bats. According to these conditions, foraging areas of bats or habitats have been defined (Aldridge and Rautenbach, 1987; Neuweiler, 1989; Fenton, 1990; Schnitzler and Kalko, 1998, 2001; Schnitzler et al., 2003; Denzinger and Schnitzler, 2004). The definitions differ partially but all approaches have in common that they separate three main types of foraging areas which Fenton (1990) named open, edge and closed habitats (for review see Schnitzler et al., 2003). We will use the terms open, edge and narrow space as first proposed by Schnitzler et al. (2003).

In our definition habitat is not just the place where an animal lives. We follow Krausman's review (1999) and define that a foraging habitat is determined by the resources and conditions which a species encounters when searching for food. This functional definition implies that species forage in the same habitat as long as they have to perform similar tasks to exploit similar resources under similar conditions. The spatial extend of such a functionally defined habitat is species-specific.

Our habitat definition is based solely on the sensory abilities of bats to perform habitat-specific tasks. Habitats differ according to the spatial relations between bat and background or food and background. The proximity of a bat to the food items and to background objects poses also a motor task (Fenton, 1990). Bats foraging in the open fly long distances with high speed and gleaning bats maneuver close to the background to get the food while also avoiding collisions. Therefore, not only the sensory system has been adapted to habitat specific tasks but also the motor system (Aldridge and Rautenbach, 1987; Norberg and Rayner, 1987; Fenton, 1990; Norberg, 1994).

**Figure 2:**
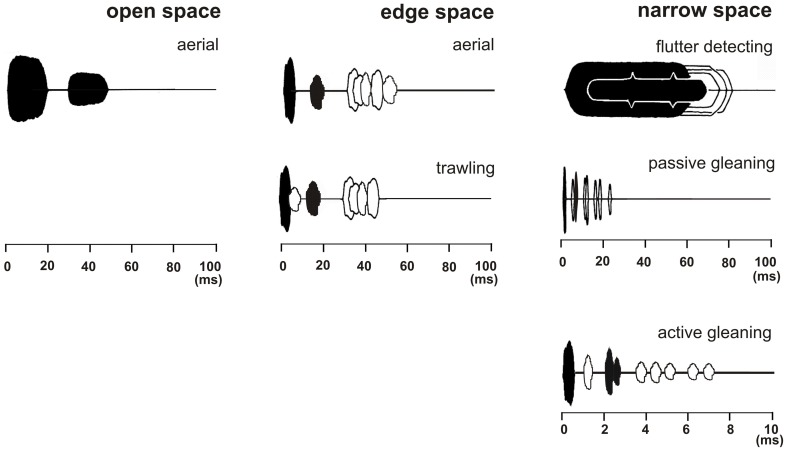
**Echolocation scenes of bats that search for prey in three different foraging habitats with typical foraging modes**. The emitted signal (black) and the returning echoes from prey (black) are displayed together with echo trains from background targets (white). In the depicted echolocation scene which covers a time range of 100 ms a bat foraging in open space in the aerial mode perceives a pulse-echo pair consisting only of the emitted signal and the returning echo (both in black) as long as the background is further away than 17 m. Bats foraging in edge space in the aerial mode perceive a pulse-echo pair that is followed by clutter echoes from the background (in white). When foraging in the trawling mode above the water an additional surface echo returns from below immediately after signal emission (in white). In narrow space the target echo is positioned in the clutter overlap zone. Here three different foraging modes are employed. In flutter detecting foragers the echoes of the long CF-FM signals are modulated in the rhythm of the insect's wing beat and can therefore be discriminated from unmodulated background echoes. Passive gleaning foragers use very short signals. They have no chance to find the food echo (black) between the clutter echoes (white) and they rely on other senses for the detection and localization of the food item. Active gleaners exploit favorable short range favorable echolocation situations where the food echo is isolated enough or is so conspicuous that it can be found between clutter echoes.

According to clutter conditions we define three types of foraging habitats which are developed from former definitions of Aldridge and Rautenbach (1987); Neuweiler (1989); Fenton (1990); Schnitzler and Kalko (1998, 2001); Schnitzler et al. (2003) and Denzinger and Schnitzler (2004) (Figure 2).

### Open space

Bats foraging in “open space” exploit the resource of airborne insects flying far from background targets and catch their prey in the *“aerial”* mode (Figure 2). Under these conditions echoes from the background reach the bat considerably later than the echoes from the prey and do not disturb their detection. In open space bats do not react to the background in their echolocation behavior.

### Edge space

Bats foraging in “edge space” exploit the resource of airborne prey found near the edges of buildings and vegetation, in gaps, or above the ground and water surfaces, and catch their prey in the *“aerial”* mode (Figure 2). Under these conditions the pairs of emitted signal and prey echo are followed by background echoes. As long as the background echoes do not overlap with the prey echoes, no masking of the prey echo occurs. In edge space bats react to the background in their echolocation behavior.

A special edge space condition is used by bats that exploit the resource of prey which is found on or just above calm water surfaces. Foraging bats fly low over water and emit their signals in forward direction. Their sound waves propagate in the air above water and partly come back as direct echoes if they hit prey or a background target. However, most of the emitted waves and of the returning echoes hit the mirror-like water surface. These waves are reflected away. Only the waves which hit the water perpendicularly, direct below the bat, produce a strong echo. The two-way travel time of this echo from below encodes the flight height of the bat and indicates water (Greif and Siemers, 2010). As trawling foragers fly low over water, the surface echo appears first and often overlaps with the emitted signal. Echoes from prey ahead of the bat appear later. Echoes from surface prey always contain a direct and a reflected component. The overall amplitude of this combined echo is larger than the direct echo produced by the same target in air due to the additional mirrored echo (Siemers et al., 2001, 2005). Background targets such as the shore produce an additional echo complex after the prey echo so that the auditory scene is similar to the situation in edge space. If background targets are far away, e.g., if the bat flies in the middle of a lake, even an open space-like auditory scene may occur, but with the important difference that the emitted signal is always followed by the surface echo from below (Figure 2).

### Narrow space

Bats foraging in “narrow space” exploit either animal prey which is positioned on or near background objects like vegetation or the ground, or they forage for fruits and flowers which are part of the background. Food echoes from animals either overlap with or are so close to background echoes that they are masked. Food echoes from plants must be discriminated from other background echoes. In narrow space bats have difficulties to find food echoes between clutter echoes only by echolocation. Three different foraging strategies have been evolved to cope with this problem.

#### Flutter detecting mode

Some bats specialize in finding their food using the “flutter detecting” foraging mode. They recognize insect echoes from their long CF-FM signals, which are modulated in the rhythm of the beating wings, and discriminate them from unmodulated clutter echoes (Figure 2).

#### Passive gleaning mode

Other bats have no chance to find the food echo in the dense clutter echoes from the background. They have to rely on other senses and use food generated cues to find it. They operate in the “passive gleaning” mode (Figure 2).

#### Active gleaning mode

Some bats are still able to find food, which is either part of the substrate or positioned on substrate, only by echolocation even under challenging clutter conditions. They forage in the “active gleaning” mode. Active gleaners use their echolocation system to exploit on short range favorable echolocation situations. Either they profit from food echoes that are isolated enough in time such that they can be identified between the clutter echoes (Figure 2), or they search for conspicuous food echoes, e.g., from flowers and fruits that can be discriminated from clutter echoes.

## Borders between foraging habitats

So far we have defined three foraging habitats where bats exploit different resources and perform different echolocation tasks. However, we have not yet defined the borders between them. The distances between bat and background and between food item and background have been identified as the most relevant ecological constraint which have shaped the foraging and echolocation behavior of bats. These distance-dependent effects have been used to define the borders between habitats (Schnitzler and Kalko, 1998, 2001; Schnitzler et al., 2003; Denzinger and Schnitzler, 2004).

**Figure 3:**
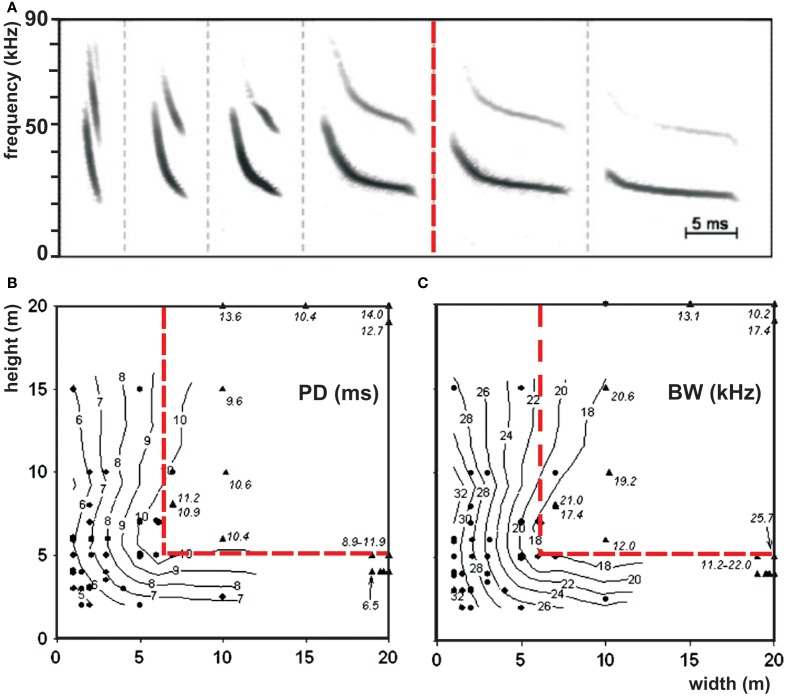
**Border between open and edge space. (A)** Repertoire of the echolocation signals of *Vespertilio murinus* while foraging in open (right to the red line) and in edge space (left to the red line) and **(B)** isocontour plots of the signal parameters pulse duration and **(C)** bandwidth as a function of the horizontal and vertical distances to the background. Each dot represents the mean value of a sequence which was emitted at the indicated position. The red line separates open space from edge space according to our definition that bats react to the background in edge space by changing signal structure but not in open space [adapted from Schaub and Schnitzler (2007)].

**Figure 4:**
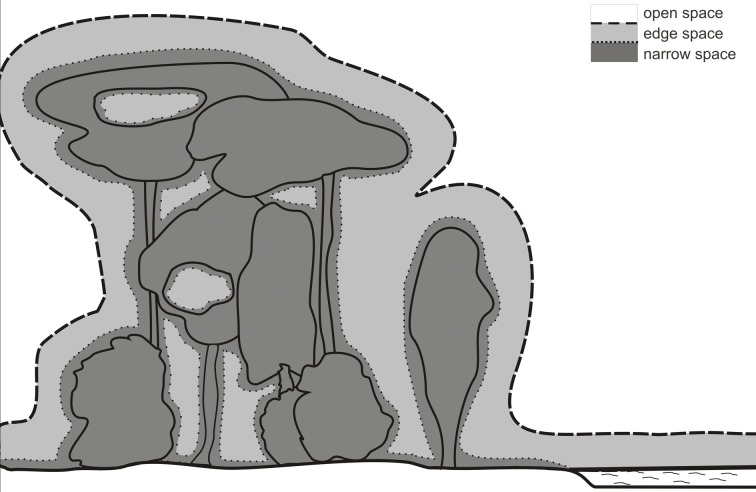
**Foraging habitats of bats**. The borders between open and edge space is determined by the echolocation behavior of the bats. Bats react to background targets in edge space but not in open space. The border is species specific. The narrow space begins with the clutter overlap zone which depends on signal duration.

The border between open and edge space is indicated by the bats' echolocation behavior (Figure 4). In open space bats do not react to the background, whereas in edge space they do. We hypothesize that in edge space bats react in their echolocation behavior to collect information necessary to maneuver in relation to background objects and to avoid collisions. The border between open and edge space is species-specific. *Vespertilio murinus* varied signal structure systematically in relation to the background. Above 6 m in horizontal direction and 5 m in vertical direction from the background, bats did no longer change their signal structure. According to our definition, this switch indicates the border between open and edge space (Schaub and Schnitzler, 2007) (Figure 3). Data from other species also show such a border. In *Pipistrellus kuhlii* the border was found at a height of about 5 m, in *Pipistrellus pygmaeus* at 3 m, and in *Eptesicus serotinus* and *Eptesicus nilssonii* at about 8–10 m (Kalko and Schnitzler, 1993; Rydell, 1993; Jensen and Miller, 1999). The species-specific spatial extend of the edge space may reflect the ability of the different species to maneuver near background objects. Fast flying bats with a lower maneuverability need more space for collision avoidance than bats which fly slower and have broader wings that equip them better for obstacle avoidance.

The border between edge and narrow space has been defined by the relation between food echo and clutter echoes from the background (Schnitzler and Kalko, 1998, 2001; Schnitzler et al., 2003; Denzinger and Schnitzler, 2004). This definition implies that a bat is in narrow space if the food item is positioned in the clutter-overlap zone where background echoes overlap with the food echo. A better definition for narrow space would be if the food echo is masked by the clutter echoes. For example, shallow modulated narrowband signals have a stronger masking effect and a wider masking zone than steeply modulated broadband signals of the same duration. However, it is very difficult to determine the exact extension of the masking zone. For practical reasons, we therefore define that the narrow space begins with the clutter overlap zone (Figure 4).

## Bat guilds

The guild concept opened the way to classify the highly diverse foraging and echolocation behaviors of microchiropteran bats by attributing species which perform similar tasks and share similar adaptations to guilds. These guilds were first defined by habitat type (uncluttered, background-cluttered, and highly-cluttered space), foraging mode (aerial-hawking, trawling and gleaning) and diet (insectivore, piscivore, carnivore, sanguivore, frugivore, nectarivore, omnivore) (Schnitzler and Kalko, 1998, 2001). In a second attempt the terms for the three habitat types were changed to the more neutral terms open, edge and narrow space to avoid misinterpretations concerning the role of background echoes in the echolocation process. Background echoes are not only disturbing clutter, but they also carry relevant information which is used for biotope and landmark recognition, and collision-free maneuvering. Additionally, diet was no longer used to classify guilds because echolocation and foraging behavior are mainly influenced by habitat type and foraging mode but not by the prey type. However, by that time it was not yet known that there are bats which find their food in narrow space by using the active gleaning mode so that only 5 guilds were defined (Schnitzler et al., 2003). Later an additional guild was added taking into consideration that some bats operate in narrow space in the active gleaning mode (Denzinger and Schnitzler, 2004). Here we will introduce a further guild that comprises all nectar, pollen and fruit eaters because these bats use the passive and the active mode to find their prey. Thus, we propose that 7 guilds are sufficient to structure even the most diverse bat communities.

## Open space aerial foragers

**Figure 5:**
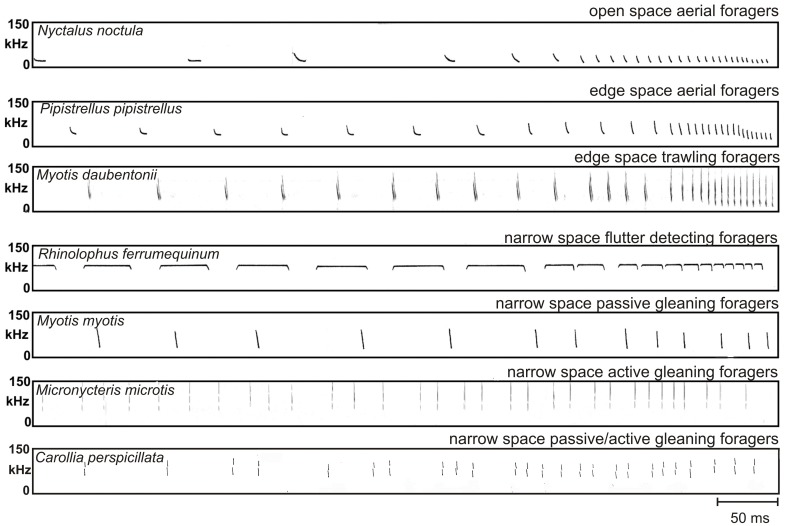
**Search and approach signals of a representative species from each guild**. The approach sequences of open space and edge space foragers end with a terminal group consisting of buzz I and buzz II. Narrow space flutter detecting foragers maintain the CF-component of the calls even in the shortest signals of the terminal group. The approach sequences of all other narrow space gleaning foragers lack a distinct terminal group. The approach signals of narrow space passive gleaners are often arranged in groups, but grouping is less distinct and pulse intervals are larger than in active and passive/active gleaning foragers. Echolocation is exclusively used for landing control. The approach signals of narrow space active and passive/active gleaning foragers are clearly arranged in groups of two to five. Repetition rate is higher than in passive gleaning foragers. Echolocation is used to approach a stationary identified food item and to evaluate the orientation of the prey in order to grab it.

Bats that hunt for airborne prey in open space face the problem that their prey is often distributed over large spaces and is therefore difficult to find. Bats that have to cope with this echolocation task are assigned to the guild of “open space aerial foragers.” They have evolved echolocation systems for long range detection of prey. They use narrowband and shallowly modulated search signals with rather long call durations of about 8 ms to 25 ms. The long and narrowband signals increase the probability to detect an insect echo, as the signal energy of the echo is concentrated for a substantial time in the corresponding neuronal filters within the auditory system. Additionally, long signals increase the chance to perceive glints in insect echoes, which are short amplitude peaks generated by the fluttering wings in the instant when the wing is perpendicular to the impinging sound waves (Schnitzler, 1987). The frequency of the relevant harmonic of the narrowband echolocation calls is generally below 30 kHz and the calls are often emitted only every second or third wing beat resulting in long pulse intervals (Figure 5). The average source levels range between 104 and 111 dB SPL calculated for 1 m in front of the bat's mouth (re 1 m) (Holderied and von Helversen, 2003). The low frequencies and high source levels guarantee large detection ranges. For example, estimations of maximum detection distances for *Nyctalus noctula*, a typical open space bat from Europe, range from 10 to 3.5 m for insects with target strengths between −40 and −65 dB (Stilz and Schnitzler, 2012). In open space foragers maximum detection distances for flying insects beyond 20 m to 25 m are very unlikely even under the most favorable conditions with low signal frequency, high emission SPL, optimal beam width, high target strength and optimal temperature and humidity (Holderied and von Helversen, 2003; Jung et al., 2007; Stilz and Schnitzler, 2012; Jakobsen et al., 2013).

The echoes of the long distance echolocation signals of open space bats also deliver information that can be used for navigation and for biotope recognition. According to Stilz and Schnitzler (2012), *N. noctula* is able to perceive echoes from a forest edge up to a maximal distance of 37 m and from a water surface up to 54 m. Open space bats do not react to the background in their echolocation behavior. This may indicate that they do not need to adjust their flight maneuvers in relation to the background.

After the detection of prey all open space foragers start with an approach sequence where pulse interval and pulse duration are reduced and signal bandwidth is increased with decreasing distance to prey. The approach sequence always ends with a distinct terminal group consisting of buzz I and buzz II. In buzz I pulse interval is further reduced, buzz II is characterized by a minimal and constant pulse interval of approximately 6 ms, and in some species also by a lower signal frequency (Figure 5). Open space aerial foragers are mainly found in the families of Rhinopomatidae, Emballonuridae, Vespertilionidae and Molossidae.

## Edge space aerial foragers

Bats that hunt for airborne prey in edge space have to find their food in the vicinity of background targets. In parallel they have to determine their own position, adjust their flight path and flight maneuvers in relation to the background, and avoid collisions. Additionally, they have to collect the information necessary for biotope recognition. These bats are assigned to the guild of “edge space aerial foragers.” To perform these echolocation tasks, edge space foragers use mixed search signals containing a shallowly modulated narrowband component preceded and/or followed by a broadband, steeply downward frequency-modulated component. The signals have an intermediate duration of about 3–10 ms and are emitted every wing beat or, if bats fly close to the background, in groups of two signals. The frequency of the shallowly modulated component is species-specific and mostly between 30 and 60 kHz in the relevant harmonic. The shallowly modulated part is suited for the detection of insects at intermediate distances, i.e., between 1.5 and 7 m (Stilz and Schnitzler, 2012). The broadband and steeply modulated signal component is suited to localize and classify background targets. Thus, it is most likely used to control maneuvers in the vicinity of background objects, including obstacle avoidance. The source levels of edge space aerial foragers are somewhat lower than those of bats that forage in open space and range from 101 to 107 dB SPL re 1 m (Holderied and von Helversen, 2003; Surlykke and Kalko, 2008).

Bats change the structure of their signals when they come closer to the background (Schaub and Schnitzler, 2007). Bandwidth is increased, duration is reduced (Figure 3) and often two signals per wing beat are emitted to increase the update rate. The reduction of signal duration keeps the overlap-free window open (Kalko and Schnitzler, 1993) and a higher sweep rate resulting from a shortening of the signal duration and an increase in bandwidth additionally increases the localization accuracy. At least for some species it has been shown that they also reduce the emission SPL when they approach the background (Surlykke and Kalko, 2008; Brinkløv et al., 2010). The approach sequences of edge space bats also end with a terminal group consisting of buzz I and buzz II (Schnitzler et al., 1987; Denzinger et al., 2001; Ratcliffe et al., 2011). Edge space aerial foragers are mainly found in the families of Emballonuridae, Mormoopidae, Vespertilionidae, and Mollossidae.

## Edge space trawling foragers

Bats belonging to the guild of “edge space trawling foragers” are found in at least three bat families: Vespertilionidae [*Myotis adversus* (Thompson and Fenton, 1982), *Myotis albescens* (Kalko et al., 1996), *Myotis daubentonii* (Kalko and Schnitzler, 1989), *Myotis dasycneme* (Britton et al., 1997), *Myotis capaccinii* (Kalko, 1990), *Myotis vivesi* (Blood and Clark, 1998), *Myotis ricketti* (Ma et al., 2007)], Noctilionidae [*Noctilio leporinus* (Schnitzler et al., 1994), *Noctilio albiventris* (Kalko et al., 1998)] and Phyllostomidae [*Macrophyllum macrophyllum* (Weinbeer and Kalko, 2007)]. Trawling foragers fly at low height above water. They either hunt for insects drifting on or flying just above calm water surfaces or for fish. Fish is detected either directly when it jumps out of the water or by the water drops arising when the fish breaks through the water surface. The sound waves that hit the water are reflected away from the bat except for those that hit the water surface in a perpendicular way, right below the bat. This echo encodes the flight height. When trawling bats hunt for prey in the vicinity of the shore they encounter similar echolocation scenes as edge space aerial foragers. On clean water surfaces the isolated prey echo is followed by the background echoes from the shore. Edge space trawling foragers have difficulties to detect prey if the water is turbulent or covered with ripples (Frenckell and Barclay, 1987; Rydell et al., 1999; Warren et al., 2000) or if plants or debris is floating on the water surface (Boonman et al., 1998). In this case the prey echo is hidden in clutter echoes (Siemers et al., 2001). If trawling bats search for prey far away from the shore, e.g., on a lake, the echolocation scene may even be similar to that of open space bats.

In search flight *Myotis* species emit mixed signals which contain steeply modulated components with a more shallowly modulated component in between. The species-specific peak frequencies of the shallowly modulated components are between 30 and 60 kHz. The signals have an intermediate duration of 3–7 ms and either one or two signals per wing beat are emitted (Kalko and Schnitzler, 1989; Jones and Rayner, 1991; Britton et al., 1997) (Figure 5). *M. macrophyllum* emits multiharmonic signals. The main energy is in the second or third harmonic with frequencies above 50 kHz. Signals have an intermediate duration of 2–4 ms (Brinkløv et al., 2010). *N. leporinus* and *N. albiventris* use a combination of pure CF-signals and mixed signals with a CF-component that is followed by a frequency modulated component. The species-specific constant frequencies are 55 and 70 kHz, respectively. The signals are usually emitted in groups. When flying low over water, the signal duration is around 6 ms but can reach up to 21 ms in *N. albiventris* when flying in high search flight (Schnitzler et al., 1994; Kalko et al., 1998). The source levels of edge space trawling foragers recorded in the field vary somewhat between species. In *M. daubentonii* the mean source level was about 100 dB SPL re 1 m (Surlykke et al., 2009) whereas *N. leporinus* and *N. albiventris* cry out much louder and reach maximal mean source levels of around 116 dB SPL re 1 m (Surlykke and Kalko, 2008). In *M. macrophyllum* the mean source level depends on the distance to background and is 85 dB SPL re 1 m in a semi-cluttered condition and 91 dB SPL re 1 m in a more open situation (Brinkløv et al., 2010). The approach sequences of all trawling *Myotis* species end with a distinct terminal group consisting of buzz I and buzz II (Figure 5). In *M. macrophyllum* the pulse interval is continuously reduced down to 6 ms between the last calls (Weinbeer and Kalko, 2007), a typical value for buzz II in other species. In *Noctilio* the CF component is given up in the terminal group, which distinguishes the Noctilionids from Rhinolophids and Hipposiderids (Schnitzler et al., 1994; Kalko et al., 1998; Übernickel et al., 2013).

Edge space trawling foragers show several morphological adaptations to the trawling mode. The hind legs and interfemoral pouches are highly specialized to take prey from the water surface or out of the water. Piscivorous species have sharp claws.

## Narrow space flutter detecting foragers

All bats that search for prey in narrow space face the problem that the prey echoes are hidden in background echoes. Bats which belong to the guild of “narrow space flutter detecting foragers” have evolved specific adaptations to overcome this problem. They use echolocation to find their prey and evaluate flutter information in the echoes of their long CF-FM signals which is encoded in a pattern of distinct amplitude and frequency modulations produced by the moving wings of the prey. The modulations are analyzed in a highly specialized hearing system with an auditory fovea. Flutter detecting foragers compensate the Doppler shifts generated by their own flight speed to keep the echo frequency within the frequency range of the auditory fovea [reviewed in Schnitzler and Denzinger (2011)]. Flutter information not only facilitates the detection but also contains information about species, size, and aspect angle of the prey (von der Emde and Menne, 1989; von der Emde and Schnitzler, 1990; Roverud et al., 1991). The short terminal FM component of the CF-FM signals is well-suited to localize background targets and the CF additionally contains flow field information that bats might use to commute along landscape contours (Müller and Schnitzler, 1999; Schnitzler et al., 2003).

Flutter detection has been evolved at least twice, in Rhinolophids and Hipposiderids and in one species of Mormoopids, *Pteronotus parnellii*. Narrow space flutter detecting foragers either hunt on the wing or from perches in the flycatcher style. Fluttering prey flying close to vegetation or sitting on surfaces is either caught in the aerial mode or gleaned from surface. In search flight signal duration in Hipposiderids is around 5–20 ms, in *P. parnellii* around 15–35 ms and in Rhinolophids around 50–80 ms. Rhinolophids mostly emit one call per wing beat, whereas *P. parnellii* often emits groups of two and Hipposderids groups with more signals. The long signal duration accounts for the very high duty cycles in narrow space flutter detecting foragers. Therefore, these bats have also been classified as “high duty cycle bats” (Neuweiler and Fenton, 1988; Fenton, 1995). The CF frequency is species-specific and ranges from about 28 kHz in *Rhinolophus paradoxolophus* to 213 kHz in *Cleotis percivali*. The approach sequence ends with a distinct terminal group. All bats of this guild have in common that the CF component is always maintained even in the shortest signals of the terminal group (Figure 5).

## Narrow space active gleaning foragers

Bats that search for food positioned on or near background objects (e.g., an insect) or which is part of the background (e.g., a fruit or a flower) face the problem that the food echoes are hidden in clutter echoes. If they use only echolocation to solve this problem, they are assigned to the guild of “narrow space active gleaning foragers.” So far only one insectivorous bat species has been identified to be a strict active gleaner that finds the prey by echolocation alone. *Micronycteris microtis*, a phyllostomid bat, forages for stationary prey items like dragon flies that sit on large leaves (Geipel et al., 2013; own unpublished data). When searching for prey *M. microtis* explore one leaf after another by approaching them oblique from above. Within about one third of a second the bats decide whether a leaf is empty. From an empty leaf the bat receives an echo train with a clutter echo from the frontal part of the leaf and an echo train with many clutter echoes from the end of the leaf and from objects behind it. All sound waves hitting the flat surface of the leaf are reflected away from the bat. Echo trains from empty leaves therefore only contain the clutter pattern without an insect echo in between, whereas leaves with prey produce an isolated additional echo between the clutter echoes (Figure 2). Active gleaning from a flat surface thus somehow resembles the echolocation scene in the trawling mode but on a micro time scale. In both situations a flat surface reflects the sound waves away from the bat so that the echoes from prey sitting on this surface stick out if the echolocation signals are short enough. When the bat has detected a leaf with prey it hovers on the spot or backward before it makes the final approach flight. When searching for prey bats emit multi-harmonic, ultra-short (0.2 ms), broadband and high-frequency calls with low amplitude. The signals are arranged in groups. The terminal group just before the prey is grasped contains 3–5 signals. A distinct buzz is missing (Figure 5).

## Narrow space passive gleaning foragers

Bats that encounter echolocation scenes, where the echo train does not deliver enough information to distinguish between food and background echoes, rely on prey generated cues alone to find their food. These bats are assigned to the guild of “narrow space passive gleaning foragers.” Animal eating passive gleaners feed on substrate bound prey such as insects, other arthropods, and small vertebrates and rely on prey generated sounds to localize the site with prey (Schmidt et al., 2000; Arlettaz et al., 2001; Goerlitz et al., 2008; Page and Ryan, 2008). Under favorable conditions vision may also play a role in prey detection (Bell, 1985; Eklöf and Jones, 2003).

After getting alerted bats approach the prey site which is indicated by prey generated cues with sufficient accuracy. Echolocation is only used to guide the approach to the site with prey. After landing on the prey bats use mainly tactile and olfactory cues to find the prey (Kolb, 1958). Under very favorable conditions passive gleaners are able to make the transition to active gleaning, e.g., if the prey is offered on a flat surface which produces no clutter echoes. So far, this transition has been demonstrated only in the laboratory (Marimuthu et al., 1995; Schmidt et al., 2000; Flick, 2008).

All animal eating narrow space passive gleaning foragers operate with short, broadband signals with low source levels. Often two to three signals are emitted within the rhythm of the wing beat. The signals are suited for spatial orientation including obstacle avoidance and biotope recognition. During the approach to the site with food, repetition rate is increased and signals are arranged in more or less distinct groups. The terminal group contains only a few signals. This echolocation pattern is typical for the approach to a landing site (Figure 5). Narrow space passive gleaning foragers are found in Phyllostomidae, Megadermatidae, Nycteridae, and Vespertilionidae.

## Narrow space passive/active gleaning foragers

Frugivorous and nectarivorous bats feed on fruits and nectar of bat-pollinated flowers. These targets are part of the background and their echoes have to be found between the echoes of other background targets. Fruits and flowers advertise their nature and position by species-specific odor bouquets but also by a specific position in relation to the background. Often also specific reflection properties result in food-specific conspicuous echoes (von Helversen and von Helversen, 1999, 2003; von Helversen et al., 2003; Simon et al., 2006, 2011). There is evidence that fruit and nectar eating bats use odor for a rough localization of the food source in the passive mode and echolocation for the precise localization in the active mode. Therefore, we assign all frugivorous and nectarivorous bats to a new guild called “narrow space passive/active gleaning bats.”

Field studies in frugivorous and nectarivorous bats clearly show that odor is the primary cue that attracts the bats (Rieger and Jakob, 1988; Laska, 1990; Hessel and Schmidt, 1994; Thies et al., 1998; von Helversen et al., 2000; Mikich et al., 2003; Korine and Kalko, 2005). Odor can be detected over long ranges, and guides the bats close to where the food is located. However, the localization accuracy for an odor source is not very high so that bats probably cannot home in on the food only by olfactory cues. Bats therefore have to switch from the odor-guided and rather imprecise passive mode to the echolocation-guided and far more precise active mode for food localization.

The precise localization of a food source by echolocation is facilitated by specific positions and properties of bat plants and flowers. For example, *Gurania spinulosa*, a flaggelichorous curcubit, exposes its cucumber shaped fruits on pendulous leafless branches in vegetation gaps. In a flight tent *Phyllostomus hastatus* not only approached the ripe fruits with the typical odor but also fruit models without odor if they were offered at the correct position. This approach was guided only by echolocation and would therefore fulfill the condition for active gleaning (Kalko and Condon, 1998). However, the experiments also revealed that the odor of ripe fruit in combination with the proper fruit position on pendulous branches is the most effective stimulus combination to evoke a response in bats. This suggests that odor also plays an important role under natural conditions. An odor- and echolocation-guided approach to food was also described for *Carollia* species approaching piper fruits (Thies et al., 1998) and for *Artibeus watsoni* and *Vampyressa pusilla* approaching figs (Korine and Kalko, 2005).

The precise localization of a food source by echolocation is also facilitated if the echo of a food item has characteristic echo properties and differs from other background echoes. Ensonification experiments have shown that a specific disc-shaped leaf or petal on the inflorescences of some bat-pollinated plants produced spatially invariant echoes with a characteristic spectral and amplitude pattern over a wide range of sound incidence angles. These conspicuous echoes are rather loud and stick out between spatially more variable background echoes (von Helversen and von Helversen, 1999; von Helversen et al., 2003; Simon et al., 2006, 2011). Behavioral studies have shown that bats use such echo beacons to localize flowers among other background echoes. The presence of a disk-shaped model leaf reduced the search time for an artificial feeder by 50% in *Glossophaga soricina* (Simon et al., 2011) and flowers were less visited if the echo producing structures were manipulated (von Helversen and von Helversen, 1999). However, in another approach von Helversen et al. (2000) showed that odor is a very important cue which attracts species of the genus *Glossophaga* to bat-pollinated flowers. They concluded for nectarivorous bats that the sense of smell plays an important role in searching for and localizing bat-pollinated flowers.

So far all studies with frugivorous and nectarivorous bats have shown that the passive and rather imprecise localization of food with odor as well as the active and precise localization of food with echolocation play a role in the foraging process. The degree of overlap between the two modes and their relative importance for the foraging process may differ between species. Our attribution does not exclude the possibility that under favorable conditions only odor or only echolocation can guide a species successfully to their food sources.

The echolocation signals of narrow space passive/active foragers are short, multi-harmonic and broadband. They have high frequencies and low source levels to reduce clutter echoes from the background. Signals are often arranged in groups and the approach sequences lack a typical buzz (Figure 5). The echolocation behavior is rather similar to that of the pure active gleaner *M. microtis* which may indicate that a stationary, rather large, identified food item is approached under the guidance of echolocation. Narrow space passive/active foragers are only found in the family of Phyllostomidae.

In theory, there might be animal eating bats that forage in the active mode and also use olfactory cues from prey to get close to the site with food. So far there are no hints that bats flying and searching for food in the active mode use olfactory cues to find their animal prey. If these bats would use olfactory cues they should be assigned to the guild of narrow space passive/active gleaning foragers.

## Adaptations in wing morphology

Bats are not only adapted in their echolocation systems to where and how they forage for prey but also in their morphology (Fenton, 1990). The most obvious ecomorphological adaptation is the shape of the wings, which reflects the demands on flight performance when foraging under particular ecological conditions. Meaningful parameters that describe the size and shape of wings are wing loading, aspect ratio and shape of the wing tip (Norberg and Rayner, 1987; Norberg, 1994). Typical open space foragers have small pointed wings with high aspect ratio which give high agility. Such a wing is adapted for a fast aerial hawking flight. Edge space foragers fly slower and are more maneuverable than open space foragers. Their wings have average aspect ratios and wing loadings and rounded tips. These wings are adapted for slow inexpensive flight in the vicinity of background objects. Edge space trawling foragers have long wings and a higher aspect ratio than most other bats but have only a medium wing loading. Such a wing is adapted for economic flight above water surfaces that allows also slow flight. All narrow space bats have short and broad wings with low aspect ratios, low wing loading and often very rounded wing tips which are adaptations for high maneuverability and slow flight in confined spaces (Norberg and Rayner, 1987; Norberg, 1994). The relation between habitat specific demands on flight performance and wing morphology is obvious. However, within guilds there are many fine grained differences in wing morphology that may reflect adaptations to different niches (Dietz et al., 2006).

## Assigning bat species to guilds

Bats can be highly flexible in their habitat use and also in their foraging modes (Fenton, 1990; Schnitzler and Kalko, 1998; Denzinger et al., 2004). Bats that mainly forage in the gleaning mode in narrow space can also fly in edge space and maybe forage there in the aerial mode, and edge space aerial foragers very often also search for prey in open space. When moving from one habitat to another and when changing the foraging mode bats also change their echolocation behavior and use the habitat- and mode-specific signal types and sound patterns. For example, aerial-hawking pipistrelles switch from more broadband mixed search signals in edge space to longer pure narrowband signals in open space (Kalko and Schnitzler, 1993). However, there are limits to the behavioral flexibility which are mainly determined by the motor capabilities of the bats (Schnitzler and Kalko, 1998). Typical open space foragers such as *Tadarida* species always forage in open space as their habitat-specific wing morphology is not suited for maneuvering near background targets. Most edge space aerial foragers do not have the motor abilities to maneuver in close vicinity to background objects necessary to exploit resources in narrow space. The access of a species to a more open habitat type is possible, but not the reverse (Fenton, 1990). Despite the behavioral flexibility found in some bats they can also be assigned to a specific guild according to their dominant foraging behavior for which their echolocation system and their wing morphology are best adapted.

The criterion for the attribution of bats to the guild of narrow space flutter detecting foragers is the use of long CF-FM echolocation signals for flutter detection and the compensation of Doppler shifts. All flutter detecting foragers maintain the CF component in all behavioral situations even in the shortest signals of the terminal group of the approach. *Noctilio* species and some smaller mormoopids sometimes also use CF-FM search signals. However, they switch to pure FM signals when approaching prey. Additionally, they do not have a sharply tuned auditory fovea and a sophisticated Doppler shift compensation system (Schnitzler and Denzinger, 2011). Therefore, we do not classify them as flutter detecting foragers.

Narrow space foragers are attributed to the guild of passive gleaning foragers if they find the preferred food source only based on passive cues. Bats that find their food relying only on echolocation are assigned to the guild of active gleaning foragers. In critical tests for the attribution to one of the guilds, passive gleaning foragers should approach a loudspeaker with playback signals from the prey, and active gleaning foragers should approach a stationary silent and non-smelling insect dummy on a leaf.

In this paper we propose a new guild of “narrow space passive/active foragers” that comprises all frugivorous and nectarivorous bats. Most bat fruits and flowers advertise their presence and position by species-specific odor bouquets as well as by specific reflection properties which produce a conspicuous echo. In their typical foraging pattern frugivorous and nectarivorous bats use both, odor and echolocation information, to find their food. We are aware that under favorable conditions odor alone or echolocation alone can guide bats to their food.

Some species are highly variable in their use of foraging modes and diets which makes it difficult to assign them to a specific guild. For example *Phyllostomus hastatus* “glean a wide variety of animal and vegetable food” (Kalko et al., 1996). They feed on insects and small vertebrates as well as on nectar, pollen, and fruit. Most likely, they use the passive/active mode for fruit and nectar acquisition, reason why we attribute *P. hastatus* to the guild of narrow space passive/active foragers.

With the guild concept we group together species that live under similar ecological conditions, perform similar tasks, and share similar sensory and motor adaptations. The foraging and echolocation behaviors of all members of a guild are so similar that the observed behavioral patterns of well-investigated species have a high predictive value for other less studied species of the same guild (Figure 5).

## Niche differentiation within guilds

Bat species from different guilds differ in their foraging behavior and in the environmental resources they use. Therefore, they do not compete for food even if they belong to the same genus. An example is *Myotis nattereri*, an edge space aerial-hawking forager, and *Myotis bechsteinii*, a narrow space passive gleaning forager. The diets of the two species differ significantly, reflecting the differences in the location where they search for prey and how they find it (Siemers and Swift, 2006).

In contrast, sympatric living species that belong to the same guild exploit similar resources and show rather similar foraging and echolocation behaviors. The members of a guild encounter the same possibly limited food resources and may face the problem of how to avoid competition. Sympatric living bats within a guild should therefore differ in at least one niche dimension. Niche differentiation can be achieved by several mechanisms such as differences in echolocation performance, sensory and cognitive abilities, maneuverability and other adaptations of the motor system, spatial segregation of foraging areas, and biogeography.

**Figure 6:**
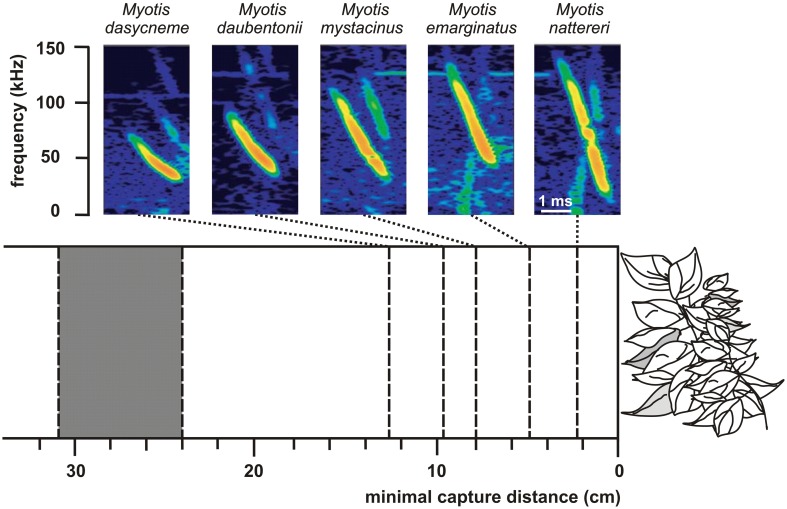
**Search call structure in relation to minimal capture distance (success rate 50%) in 5 sympatric *Myotis* species**. The higher the signal bandwidth of a species the lower is the minimal capture distance for suspended mealworms. The gray block between 24 and 31 cm indicates the range of the outer borders of the clutter overlap zones of the five bats as calculated from the sound durations of the signals. Note that the performance which is an indicator for the masking effect of the clutter echoes strongly depends on signal structure [data from Siemers and Schnitzler (2004)].

Differences in echolocation behavior especially in signal frequency but also in duration and bandwidth may account for niche partitioning within a guild (Denzinger et al., 2004; Siemers and Schnitzler, 2004). With decreasing frequency the maximum detection distance increases and directionality decreases (Stilz and Schnitzler, 2012). Thus, frequency has a huge effect on the search volume of bats which strongly increases with decreasing frequency. Signal frequency also determines the target strength of prey which depends on the relationship between the wavelength of the echolocation signal and target size. If the wing length of a prey insect is around and below the wavelength of the echolocation signal the target strength is reduced by Raleigh scattering (Houston et al., 2004). At a signal frequency of 10 kHz the critical Raleigh region is reached for wing lengths below 34 mm and at a frequency of 100 kHz for wing lengths below 3.4 mm. Bats operating with lower frequencies thus have a lower detection probability for small insects which may result in resource partitioning between sympatric species. Shi et al. (2009) present data which support this hypothesis. They compared two CF-bats with similar size but different CF-frequency and found that *Rhinolophus macrotis*, a low-frequency horseshoe bat with a CF-frequency of 57 kHz, fed in general on larger prey with wing lengths ranging from 5.2 to 37.1 mm than *R. lepidus* a high-frequency horseshoe bat with a CF-frequency of 91 kHz and wing lengths between 3.5 and 27.5 mm. Signal duration is another parameter which influences the detection probability for different sized insects. Long signals produce a wide signal overlap zone which hampers the detection of weak echoes from small insects at close range. Long signals with low frequency are mainly produced by open space foragers. Since long signals and also low frequencies reduce the probability for the detection of smaller insects in bats, Schnitzler and Kalko (1998) proposed the size filtering hypothesis. The lower the frequency and the longer the signals the larger is the just detectable prey. Bats with long signals and low frequencies are adapted for the long range detection of large insects but miss smaller ones whereas bats with shorter signals and higher frequencies have shorter detection ranges but, additionally, find insects which are smaller and fly closer to them. This general trend has been confirmed by a number of studies (e.g., Barclay, 1985, 1986; Kalko, 1995; Houston et al., 2004). The role of bandwidth in niche differentiation was demonstrated for some morphologically similar and sympatric edge space aerial/trawling species of the genus *Myotis* (Siemers and Schnitzler, 2004). The performance to detect prey in front of a clutter producing background depended on the bandwidth of the echolocation signals (Figure 6). The minimal detection distance decreased with increasing bandwidth thus indicating that differences in the echolocation system result in sensory based niche partitioning. Comparable studies with paleotropical species of the vespertilionid subfamilies Kerivoulinae and Murininae came to similar results (Schmieder et al., 2012).

There are, however, many other mechanisms besides echolocation that account for niche differentiation. Niche differentiation by spatial segregation in foraging areas has been shown for the passive gleaners *Myotis myotis* and *Myotis blythii*. While *M. blythii* depends on grassland habitats *M. myotis* selects foraging areas with access to ground-dwelling prey (Arlettaz, 1999). The spatial separation is also mirrored in the trophic niche separation of the two species (Arlettaz et al., 1997). The five species of European horseshoe bats constitute another interesting example for niche partitioning. They belong to the guild of flutter detecting foragers and have a rather similar echolocation behavior with only small differences in the species-specific frequency of the CF-FM signals. Nevertheless, they differ in foraging area, food preferences, and whether they search for fluttering prey from perches or on the wing (Dietz et al., 2007). Dietz et al. (2006) found differences in wing morphology between the species which may be just one among other mechanisms that account for the observed niche differentiation.

## Concluding remarks

Many bat communities comprise a large number of species with a high diversity in foraging and echolocation behaviors. The assignment of species living under similar constraints and performing comparable tasks into functional groups or guilds identifies patterns of community structure and helps us to understand the factors that underlie the organization of the highly diverse bat communities. Bats within each guild forage under similar ecological conditions and share comparable sensory and motor adaptations. These task-specific adaptations have a high predictive value for the assignment of bats into a guild. Habitat and foraging mode predict the echolocation behavior of a species and vice versa echolocation behavior predicts to which guild a bat can be assigned. Bat species from different guilds do not compete for food as they differ in the environmental resources they use and in their foraging behavior. However, sympatric living species belonging to the same guild often exploit the same class of resources. To avoid competition they should differ in at least one niche dimension. The fine grain structure of bat communities below the rather coarse classification into guilds is determined by mechanisms that result in niche partitioning.

### Conflict of interest statement

The authors declare that the research was conducted in the absence of any commercial or financial relationships that could be construed as a potential conflict of interest.

## References

[B1] Aldridge H. D. J. N. Rautenbach I. L. (1987). Morphology, echolocation and resource partitioning in insectivorous bats. J. Anim. Ecol. 56, 763–778 10.2307/4947

[B2] Arlettaz R. (1999). Habitat selection as a major resource partitioning mechanism between the two sympatric sibling bat species *Myotis myotis* and *Myotis blythii*. J. Anim. Ecol. 68, 460–471 10.1046/j.1365-2656.1999.00293.x

[B3] Arlettaz R. Jones G. Racey P. A. (2001). Effects of acoustic clutter on prey detection by bats. Nature 414, 742–745 10.1098/rspb.2012.2830 11742397

[B4] Arlettaz R. Perrin N. Hausser J. (1997). Trophic resource partitioning and competition between two sibling bat species *Myotis myotis* and *Myotis blythii*. J. Anim. Ecol. 66, 897–911

[B5] Barclay R. M. R. (1985). Long- versus short range foraging strategies of hoary (*Lasiurus cinereus*) and silver-haired (*Lasionycteris noctivagans*) bats and consequences for prey selection. Can. J. Zool. 63, 2507–2515

[B6] Barclay R. M. R. (1986). The echolocation calls of hoary (*Lasiurus cinereus*) and silver-haired (*Lasionycteris noctivagans*) bats and the consequences for prey selection. Can. J. Zool. 64, 2700–2705

[B7] Bell G. P. (1985). The sensory basis of prey location by the California leaf-nosed bat *Macrotus californicus* (Chiroptera: Phyllostomidae). Behav. Ecol. Sociobiol. 16, 343–347

[B8] Blood B. R. Clark M. K. (1998). Myotis vivesi. Mamm. Species 588, 1–5

[B9] Boonman A. M. Boonman M. Bretschneider F. van de Grind W. A. (1998). Prey detection in trawling insectivorous bats: duckweed affects hunting behaviour in Daubenton's bat, *Myotis daubentonii*. Behav. Ecol. Sociobiol. 44, 99–107

[B10] Brinkløv S. Kalko E. K. V. Surlykke A. (2010). Dynamic adjustment of biosonar intensity to habitat clutter in the bat *Macrophyllum macrophyllum* (Phyllostomidae). Behav. Ecol. Sociobiol. 64, 1867–1874

[B11] Britton A. R. C. Jones G. Rayner J. M. V. Boonman A. M. Verboom B. (1997). Flight performance, echolocation and foraging behaviour in pond bats, *Myotis dasycneme* (Chiroptera: Vespertilionidae). J. Zool. Lond. 241, 503–522

[B12] Crome F. H. J. Richards G. C. (1988). Bat and gaps: microchiropteran community structure in a Queensland rain forest. Ecology 69, 1960–1969

[B13] Denzinger A. Kalko E. K. V. Jones G. (2004). Ecological and evolutionary aspects of echolocation in bats, in Echolocation in Bats and Dolphins, eds Thomas J. A. Moss C. F. Vater M. (Chicago, IL: The University of Chicago Press), 311–326 10.1007/s00359-003-0406-2

[B14] Denzinger A. Schnitzler H.-U. (2004). Perceptual tasks in echolocating bats, in Dynamic Perception, eds Ilg U. J. Bülthoff H. H. Mallot H. A. (Berlin: Akademische Verlagsgesellschaft), 33–38

[B15] Denzinger A. Siemers B. M. Schaub A. Schnitzler H.-U. (2001). Echolocation by the barbastelle bat, *Barbastella barbastellus*. J. Comp. Physiol. A 187, 521–528 11730299 10.1007/s003590100223

[B16] Dietz C. Dietz I. Siemers M. B. (2006). Wing measurement variations in the five European horseshoe bat species (Chiroptera: Rhinolophidae). J. Mammal. 87, 1241–1251

[B17] Dietz C. von Helversen O. Nill D. (2007). Handbuch der Fledermäuse Europas und Nordwestafrikas. Stuttgart: Franckh Kosmos Verlag

[B18] Eklöf J. Jones G. (2003). Use of vision in prey detection by brown long-eared bats, *Plecotus auritus*. Anim. Behav. 66, 949–953

[B19] Fenton M. B. (1990). The foraging behaviour and ecology of animal-eating bats. Can. J. Zool. 68, 411–422

[B20] Fenton M. B. (1995). Natural history and biosonar signals, in Hearing by Bats, eds Popper A. N. Fay R. R. (New York, NY: Springer), 37–86

[B21] Flick J. (2008). Der Wechsel vom Passiven zum Aktiven Substratfang beim Großen Mausohr (Myotis myotis, Borkhausen 1797). Diploma thesis, University Tübingen, Tübingen.

[B22] Frenckell B. V. Barclay R. M. R. (1987). Bat activity over calm and turbulent water. Can. J. Zool. 65, 219–222

[B23] Geipel I. Jung K. Kalko E. K. V. (2013). Perception of silent and motionless prey on vegetation by echolocation in the gleaning bat *Micronycteris microtis*. Proc. R. Soc. B 280:20122830 10.1098/rspb.2012.2830 23325775 PMC3574334

[B24] Goerlitz H. R. Greif S. Siemers B. M. (2008). Cues for acoustic detection of prey: insect rustling sounds and the influence of walking substrates. J. Exp. Biol. 211, 2799–2806 10.1242/jeb.019596 18723538

[B25] Greif S. Siemers B. M. (2010). Innate recognition of water bodies in echolocating bats. Nat. Commun. 107, 1–6 10.1038/ncomms1110 21045825 PMC3060641

[B26] Hessel K. Schmidt U. (1994). Multimodal orientation in *Carollia perspicillata* (Phyllostomidae). Folia Zool. 43, 339–346

[B27] Hill J. E. Smith J. D. (1984). Bats: A Natural History. London: British Museum (Natural History).

[B28] Holderied M. W. von Helversen O. (2003). Echolocation range and wingbeat period match in aerial-hawking bats. Proc. R. Soc. Lond. B 270, 2293–2299 10.1098/rspb.2003.2487 14613617 PMC1691500

[B29] Holland R. A. Kirschvink J. L. Doak T. G. Wikelski M. (2008). Bats use magnetite to detect the earth's magnetic field. PLoS ONE 3:e1676 10.1371/journal.pone.0001676 18301753 PMC2246016

[B30] Holland R. A. Thorup K. Vonhof M. Cochran W. Wikelski M. (2006). Navigation: bat orientation using earth's magnetic field. Nature 444, 702 10.1038/444702a 17151656

[B31] Houston R. D. Boonman A. M. Jones G. (2004). Do echolocation signal parameters restrict bats' choice of prey?, in Echolocation in Bats and Dolphins, eds Thomas J. A. Moss C. F. Vater M. (Chicago, IL: The University of Chicago Press), 339–345

[B32] Jakobsen L. Ratcliffe J. M. Surlykke A. (2013). Convergent acoustic field of view in echolocating bats. Nature 493, 93–96 10.1038/nature11664 23172147

[B33] Jensen M. E. Miller L. A. (1999). Echolocation signals of the bat *Eptesicus serotinus* recorded using a vertical microphone array: effect of flight altitude on searching signals. Behav. Ecol. Sociobiol. 47, 60–69 10.1007/s002650050650

[B34] Jones G. Rayner J. M. V. (1991). Flight performance, foraging tactics and echolocation in the trawling insectivorous bat *Myotis adversus* (Chiroptera: Vespertilionidae). J. Zool. Lond. 225, 393–412 10.1111/j.1469-7998.1991.tb03824.x

[B35] Jones G. Teeling E. C. (2006). The evolution of echolocation in bats. Trends Ecol. Evol. 21, 149–156 10.1016/j.tree.2006.01.001 16701491

[B36] Jung K. Kalko E. K. V. von Helversen O. (2007). Echolocation calls in Central American emballonurid bats: signal design and call frequency alteration. J. Zool. 272, 125–137

[B37] Kalko E. (1990). Field study on the echolocation and hunting behaviour of the long-fingered bat, *Myotis capaccinii*. Bat Res. News 31, 42–43

[B38] Kalko E. K. V. (1995). Insect persuit, prey capture and echolocation in pipistrelle bats (Microchiroptera). Anim. Behav. 50, 861–880 10.1016/0003-3472(95)80090-5

[B39] Kalko E. K. V. (1997). Diversity in tropical bats, in Tropical Biodiversity and Systematics, ed Ulrich H. (Bonn: Zoologisches Forschungsinstitut und Museum König), 13–43

[B40] Kalko E. K. V. Condon M. A. (1998). Echolocation, olfaction and fruit display: how bats find fruit of flagellichorous cucubits. Funct. Ecol. 12, 364–372 12417821

[B41] Kalko E. K. V. Handley C. O. Handley D. (1996). Organization, diversity, and long-term dynamics of a neotropical bat community, in Long-term Studies in Vertebrate Communities, eds Cody M. Smallwood J. (Los Angeles, CA: Academic Press), 503–553

[B42] Kalko E. K. V. Schnitzler H.-U. (1989). The echolocation and hunting behavior of Daubenton's bat, *Myotis daubentoni*. Behav. Ecol. Sociobiol. 24, 225–238 10.1007/BF00295202

[B43] Kalko E. K. V. Schnitzler H.-U. (1993). Plasticity in echolocation signals of European pipistrelle bats in search flight: implications for habitat use and prey detection. Behav. Ecol. Sociobiol. 33, 415–428 10.1007/BF00170257

[B44] Kalko E. K. V. Schnitzler H.-U. Kaipf I. Grinnell A. D. (1998). Echolocation and foraging behavior of the lesser bulldog bat, *Noctilio albiventris*: preadaptations for piscivory. Behav. Ecol. Sociobiol. 42, 305–319 10.1007/s002650050443

[B45] Kolb A. (1958). Nahrung und nahrungsaufnahme bei fledermäusen. Z. Säugetierk. 23, 84–95

[B46] Korine C. Kalko E. K. V. (2005). Fruit detection and discrimination by small fruit-eating bats (Phyllostomidae): echolocation call design and olfaction. Behav. Ecol. Sociobiol. 59, 12–23 10.1007/s00265-005-0003-1

[B47] Krausman P. R. (1999). Some basic principles of habitat use, in Grazing Behavior of Livestock and Wildlife, eds Launchbaugh K. L. Sanders K. D. Mosley J. C. (Moscow, ID: Idaho Forest, Wildlife and Range Exp. Sta. Bull No. 70, University of Idaho), 85–90

[B48] Laska M. (1990). Olfactory sensitivity to food odor components in the short-tailed fruit bat, *Carollia perspicillata* (Phyllostomatidae, Chiroptera). J. Comp. Physiol. A 166, 395–399 10.1007/BF00204812

[B49] Ma J. Jones G. Zhang S. Shen J. Metzner W. Zhang L. (2007). Dietary analysis confirms that Rickett's big-footed bat (*Myotis ricketti*) is a piscivore. J. Zool. 261, 245–248 10.1017/S095283690300414X

[B50] McNab B. K. (1971). The structure of tropical bat faunas. Ecology 52, 353–358 10.2307/1934596

[B51] Marimuthu G. Habersetzer J. Leipert D. (1995). Active gleaning from the water surface by the Indian false vampire bat, *Megaderma lyra*. Ethology 99, 61–74 10.1111/j.1439-0310.1995.tb01089.x

[B52] Mikich S. B. Bianconi G. V. Maia B. H. L. N. S. Teixeira S. D. (2003). Attraction of the fruit-eating bat *Carollia perspicilata* to *Piper gaudichauganium* essential oil. J. Chem. Ecol. 29, 2379–2383 10.1023/A:1026290022642 14682519

[B53] Müller R. Schnitzler H. U. (1999). Acoustic flow perception in CF-bats: properties of the available cues. J. Acoust. Soc. Am. 105, 2958–2966 10.1121/1.426909 10335645

[B54] Neuweiler G. (1989). Foraging ecology and audition in echolocating bats. Trends Ecol. Evol. 4, 160–166 10.1016/0169-5347(89)90120-1 21227342

[B55] Neuweiler G. Fenton M. B. (1988). Behaviour and foraging ecology of echolocating bats, in Animal Sonar Processes and Performance, eds Nachtigall P. E. Moore P. W. B. (New York, NY: Plenum Press), 535–549

[B56] Norberg U. M. (1994). Wing design, flight performance, and habitat use in bats, in Ecological Morphology: Integrative Organismal Biology, eds Wainwright P. C. Reilly S. M. (Chicago, IL: University of Chicago Press), 205–239 10.1088/1748-3182/4/1/015001

[B57] Norberg U. M. Rayner J. M. V. (1987). Ecological morphology and flight in bats (Mammalia: Chiroptera): wing adaptation, flight performance, foraging strategy and echolocation. Philos. Trans. R. Soc. Lond. B Biol. Sci. 316, 335–427 10.1098/rstb.1987.0030

[B58] Page A. R. Ryan M. J. (2008). The effect of signal complexity on localization performance in bats that localize frog calls. Anim. Behav. 76, 761–769 10.1016/j.anbehav.2008.05.006

[B59] Ratcliffe J. M. Jakobsen L. Kalko E. K. V. Surlykke A. (2011). Frequency alternation and an offbeat rhythm indicate foraging behavior in the echolocating bat, *Saccopteryx bilineata*. J. Comp. Physiol. A 197, 413–423 10.1007/s00359-011-0630-0 21327333

[B60] Rieger J. M. Jakob E. M. (1988). The use of olfaction in food location by frugivorous bats. Biotropica 20, 161–164 10.2307/2388189

[B61] Root R. B. (1967). The niche exploitation pattern of the blue-gray gnatcatcher. Ecol. Monogr. 37, 317–350 10.2307/1942327

[B62] Roverud R. C. Nitsche V. Neuweiler G. (1991). Discrimination of wing beat motion by bats, correlated with echolocation sound pattern. J. Comp. Physiol. A 168, 259–263 10.1007/BF00218418 2046046

[B63] Rydell J. (1993). Variation in the sonar of an aerial-hawking bat (*Eptesicus nilssonii*). Ethology 93, 275–284 10.1111/j.1439-0310.1993.tb01209.x

[B64] Rydell J. Miller L. A. Jensen M. E. (1999). Echolocation constraints of Daubenton's bat foraging over water. Funct. Ecol. 13, 247–255 10.1046/j.1365-2435.1999.00304.x

[B65] Schaub A. Schnitzler H. U. (2007). Echolocation behavior of the bat *Vespertilio murinus* reveals the border between the habitat types “edge” and “open space.” Behav. Ecol. Sociobiol. 61, 513–523 10.1007/s00265-006-0279-9

[B66] Schmidt S. Hanke S. Pillat J. (2000). The role of echolocation in the hunting of terrestrial prey – new evidence for an underestimated strategy in the gleaning bat, *Megaderma lyra*. J. Comp. Physiol. A 186, 975–988 10.1007/s003590000151 11138799

[B67] Schmieder D. A. Kingston T. Hashim R. Siemers B. M. (2012). Sensory constraints on prey detection performance in an ensemble of vespertilionid understorey rain forest bats. Funct. Ecol. 26, 1043–1053 10.1111/j.1365-2435.2012.02024.x

[B68] Schnitzler H. U. (1987). Echoes of fluttering insects: information for echolocating bats, in Recent Advances in the Study of Bats, eds Fenton M. B. Racey P. Rayner J. M. V. (Cambridge: Cambridge University Press), 226–243

[B69] Schnitzler H. U. Denzinger A. (2011). Auditory fovea and doppler shift compensation: adaptations for flutter detection in echolocating bats using cf-fm signals. J. Comp. Physiol. A 197, 541–559 10.1007/s00359-010-0569-6 20857119

[B70] Schnitzler H. U. Kalko E. K. V. (1998). How echolocating bats search and find food, in Bat Biology and Conservation, eds Kunz T. H. Racey P. A. (Washington, DC: Smithsonian Institution Press), 183–196

[B71] Schnitzler H. U. Kalko E. K. V. (2001). Echolocation by insect-eating bats. Bioscience 51, 557–569 10.1641/0006-3568(2001)051[0557:EBIEB]2.0.CO;2

[B72] Schnitzler H. U. Kalko E. K. V. Denzinger A. (2004). Evolution of echolocation and foraging behavior in bats, in Echolocation in Bats and Dolphins, eds Thomas J. A. Moss C. F. Vater M. (Chicago, IL: The University of Chicago Press), 331–339

[B73] Schnitzler H. U. Kalko E. K. V. Kaipf I. Grinnell A. D. (1994). Fishing and echolocation behavior of the greater bulldog bat, *Noctilio leporinus*, in the field. Behav. Ecol. Sociobiol. 35, 327–345 10.1007/BF00184422

[B74] Schnitzler H. U. Kalko E. Miller L. A. Surlykke A. (1987). The echolocation and hunting behavior of the bat *Pipistrellus kuhli*. J. Comp. Physiol. A 161, 267–274 10.1007/BF00615246 3625576

[B75] Schnitzler H. U. Moss C. F. Denzinger A. (2003). From spatial orientation to food acquisition in echolocating bats. Trends Ecol. Evol. 18, 386–394 10.1016/S0169-5347(03)00185-X

[B76] Shi L. Feng J. Liu Y. Ye G. Zhu X. (2009). Is food resource partitioning responsible for deviation of echolocation call frequencies from allometry in *Rhinolophus macrotis*. Acta Theriol. 54, 371–382 10.4098/j.at.0001-7051.099.2008

[B77] Siemers B. M. Baur E. Schnitzler H. U. (2005). Acoustic mirror effect increases prey detection distance in trawling bats. Naturwissenschaften 92, 272–276 10.1007/s00114-005-0622-4 15871000

[B78] Siemers B. M. Schnitzler H. U. (2000). Natterer's bat (*Myotis nattereri* Kuhl, 1818) hawks for prey close to vegetation using echolocation signals of very broad bandwidth. Behav. Ecol. Sociobiol. 47, 400–412 10.1007/s002650050683

[B79] Siemers B. M. Schnitzler H. U. (2004). Echolocation signals reflect niche differentiation in five sympatric bat species. Nature 429, 657–661 10.1038/nature02547 15190352

[B80] Siemers B. M. Stilz P. Schnitzler H. U. (2001). The acoustic advantage of hunting at low heights above water: behavioural experiments on the European ‘trawling’ bats *Myotis capaccinii*, *M. dasycneme* and *M. daubentonii*. J. Exp. Biol. 204, 3843–3854 11807102 10.1242/jeb.204.22.3843

[B81] Siemers B. M. Swift S. M. (2006). Differences in sensory ecology contribute to resource partitioning in the bats *Myotis bechsteinii* and *Myotis nattereri* (Chiroptera: Vespertilionidae). Behav. Ecol. Sociobiol. 59, 373–380 10.1007/s00265-005-0060-5

[B82] Simon R. Holderied M. W. Koch C. U. von Helversen O. (2011). Floral acoustics: conspicuous echoes of a dish-shaped leaf attract bat pollinators. Science 333, 631–633 10.1126/science.1204210 21798950

[B83] Simon R. Holderied M. W. von Helversen O. (2006). Size discrimination of hollow hemispheres by echolocation in a nectar feeding bat. J. Exp. Biol. 209, 3599–3609 10.1242/jeb.02398 16943500

[B84] Stilz W. P. Schnitzler H. U. (2012). Estimation of the acoustic range of bat echolocation for extended targets. J. Acoust. Soc. Am. 132, 1765–1775 10.1121/1.4733537 22978903

[B85] Surlykke A. Kalko E. K. V. (2008). Echolocating bats cry out loud to detect their prey. PLoS ONE 3:e2036 10.1371/journal.pone.0002036 18446226 PMC2323577

[B86] Surlykke A. Pedersen S. B. Jacobsen L. (2009). Echolocating bats emit a highly directional sonar sound beam in the field. Proc. Royal Soc. B 276, 853–860 10.1098/rspb.2008.1505 19129126 PMC2664374

[B87] Thiele J. Winter Y. (2005). Hierarchical strategy for relocating food targets in flower bats: spatial memory versus cue-directed search. Anim. Behav. 69, 315–327 10.1016/j.anbehav.2004.05.012

[B88] Thies W. Kalko E. K. V. Schnitzler H. U. (1998). The roles of echolocation and olfaction in two Neotropical fruit-eating bats, *Carollia perspicillata* and *C. castanea*, feeding on Piper. Behav. Ecol. Sociobiol. 42, 397–409 10.1007/s002650050454

[B89] Thompson D. Fenton M. B. (1982). Echolocation and feeding behavior of *Myotis adversus* (Chiroptera: Vespertilionidae). Austr. J. Zool. 30, 543–546 10.1071/ZO9820543

[B90] Trullier O. (1997). Biologically based artificial navigation systems: review and prospects. Prog. Neurobiol. 51, 483–544 10.1016/S0301-0082(96)00060-3 9153072

[B91] Übernickel K. Tschapka M. Kalko E. K. V. (2013). Flexible echolocation behavior of trawling bats during approach of continuous or transient prey cues. Front. Physiol. 4:96 10.3389/fphys.2013.00096 23675352 PMC3650317

[B92] von der Emde G. Menne D. (1989). Discrimination of insect wingbeat-frequencies by the bat *Rhinolophus ferrumequinum*. J. Comp. Physiol. A 164, 663–671

[B93] von der Emde G. Schnitzler H. U. (1990). Classification of insects by echolocating greater horseshoe bats. J. Comp. Physiol. A 167, 423–430

[B94] von Helversen D. Holderied M. W. von Helversen O. (2003). Echoes of bat-pollinated bell-shaped flowers: conspicuous for nectar-feeding bats. J. Exp. Biol. 206, 1025–1034 12582145 10.1242/jeb.00203

[B95] von Helversen D. von Helversen O. (1999). Acoustic guide in bat-pollinated flower. Nature 398, 259–260

[B96] von Helversen D. von Helversen O. (2003). Object recognition by echolocation: a nectar-feeding bat exploiting the flowers of a rain forest vine. J. Comp. Physiol. A 189, 327–336 10.1007/s00359-003-0405-3 12712362

[B97] von Helversen O. Winkler L. Bestmann H. J. (2000). Sulphur-containing Ďperfumes“ attract flower-visiting bats. J. Comp. Physiol. A 186, 143–153 10707312 10.1007/s003590050014

[B98] Warren R. D. Waters D. A. Altringham J. D. Bullock D. J. (2000). The distribution of Daubenton's bats (*Myotis daubentonii*) and pipistrelle bats (*Pipistrellus pipistrellus*) (Vespertilionidae) in relation to small-scale variation in riverine habitat. Biol. Conserv. 92, 85–91 10.1016/S0006-3207(99)00062-2

[B99] Wang Y. Pan Y. Parsons S. Walker M. Zhang S. (2007). Bats respond to polarity of a magnetic field. Proc. Royal Soc. B 274, 901–2905 10.1098/rspb.2007.0904 17848365 PMC2288691

[B100] Weinbeer M. Kalko E. K. V. (2007). Ecological niche and phylogeny: the high complex echolocation behavior of the trawling long-legged bat, *Macrophyllum macrophyllum*. Behav. Ecol. Sociobiol. 61, 1337–1348

[B101] Yovel Y. Franz M. O. Stilz P. Schnitzler H. U. (2011). Complex echo classification by echolocating bats: a review. J. Comp. Physiol. A. 197, 475–490 10.1007/s00359-010-0584-7 20848111

[B102] Yovel Y. Stilz P. Franz M. O. Boonman A. Schnitzler H. U. (2009). What a plant sounds like: the statistics of vegetation echoes as received by echolocating bats. PLoS Comput. Biol. 5:e1000429 10.1371/journal.pcbi.1000429 19578430 PMC2699101

